# Individual patient data meta-analysis of diagnostic and prognostic studies in obstetrics, gynaecology and reproductive medicine

**DOI:** 10.1186/1471-2288-9-22

**Published:** 2009-03-27

**Authors:** Kimiko A Broeze, Brent C Opmeer, Lucas M Bachmann, Frank J Broekmans, Patrick MM Bossuyt, Sjors FPJ Coppus, Neil P Johnson, Khalid S Khan, Gerben ter Riet, Fulco van der Veen, Madelon van Wely, Ben WJ Mol

**Affiliations:** 1 Centre for Reproductive Medicine, Department of Obstetrics and Gynaecology, Academic Medical Centre (AMC), Amsterdam, The Netherlands; 2 Department of Clinical Epidemiology, Biostatistics and Bioinformatics, Academic Medical Centre (AMC), Amsterdam, The Netherlands; 3 Division of Epidemiology and Biostatistics, Department of Social and Preventive Medicine, University of Bern, Switzerland; 4 Department for Reproductive Medicine and Gynaecology, University Medical Centre, Utrecht, The Netherlands; 5 Department of Obstetrics and Gynaecology, University of Auckland, National Women's Health, Auckland Hospital, Auckland, New Zealand; 6 Department of Obstetrics & Gynaecology, University of Birmingham & Birmingham Women's Hospital, Birmingham, UK; 7 Department of General Medicine, Academic Medical Centre (AMC), Amsterdam, The Netherlands

## Abstract

**Background:**

In clinical practice a diagnosis is based on a combination of clinical history, physical examination and additional diagnostic tests. At present, studies on diagnostic research often report the accuracy of tests without taking into account the information already known from history and examination. Due to this lack of information, together with variations in design and quality of studies, conventional meta-analyses based on these studies will not show the accuracy of the tests in real practice. By using individual patient data (IPD) to perform meta-analyses, the accuracy of tests can be assessed in relation to other patient characteristics and allows the development or evaluation of diagnostic algorithms for individual patients.

In this study we will examine these potential benefits in four clinical diagnostic problems in the field of gynaecology, obstetrics and reproductive medicine.

**Methods/design:**

Based on earlier systematic reviews for each of the four clinical problems, studies are considered for inclusion. The first authors of the included studies will be invited to participate and share their original data. After assessment of validity and completeness the acquired datasets are merged. Based on these data, a series of analyses will be performed, including a systematic comparison of the results of the IPD meta-analysis with those of a conventional meta-analysis, development of multivariable models for clinical history alone and for the combination of history, physical examination and relevant diagnostic tests and development of clinical prediction rules for the individual patients. These will be made accessible for clinicians.

**Discussion:**

The use of IPD meta-analysis will allow evaluating accuracy of diagnostic tests in relation to other relevant information. Ultimately, this could increase the efficiency of the diagnostic work-up, e.g. by reducing the need for invasive tests and/or improving the accuracy of the diagnostic workup. This study will assess whether these benefits of IPD meta-analysis over conventional meta-analysis can be exploited and will provide a framework for future IPD meta-analyses in diagnostic and prognostic research.

## Background

Ancient Egyptian medical papyri (1550 BC) already emphasised diagnosis by physical examination as the cornerstone of the decision to treat or not to treat an ailment [1]. Today, the clinical assessment of the probability of a disease comes from a series of implicitly and explicitly performed tests. In addition to the implicit diagnostic information from history (risk factors and symptoms) and clinical examination (signs), many additional diagnostic imaging or laboratory tests are available. The accuracy of such tests requires to be appropriately assessed before they can be used in clinical practice.

Studies on primary diagnostic research typically examine the accuracy of a test isolated from history and clinical examination or do not adjust for overlap of information captured by clinical history, physical examination and additional tests. Such studies and conventional meta-analyses of their reported results will therefore not show how useful the test will be in practice [2-4].

In addition to the predominance of isolated, single test evaluations in published literature, variations in design and quality of studies on diagnostic topics [5-8] make the interpretation of test accuracy data difficult [9-12]. Systematic reviews and meta-analyses, by definition, can not overcome these difficulties [13]. Apart from intrinsic flaws in the original studies and methodological challenges in statistically pooling results [14,15], there is concern about the generalisability of results of such meta analyses, due to the invalidity of assumptions about the constancy of accuracy measures (sensitivities, specificities, and likelihood ratios) across different patient groups [16-20].

Due to the limited space in medical journals and the lack of standard procedures to make original data accessible, little empirical evidence is available about the influence of many patient and study characteristics (i.e. patients' selection criteria, spectrum of disease, frequency of indeterminate test results and of drop outs, and the degree of blinding) on the estimates of diagnostic performance of tests [13,21].

Another limitation is the fact that many original reports of diagnostic and prognostic meta-analyses report data only in a dichotomous way, since many test results that are continuous in nature are classified as abnormal or normal. By doing so, these meta-analyses are based on reduced information, thus neglecting the potential diagnostic information contained in continuous test results. They possibly give an overestimation of the accuracy by selection of optimal cut-off values in the original studies [3,22-24].

As a consequence, it is difficult to make a good assessment of the generalisability of the accuracy of tests, either in an isolated situation as well in the context of other tests.

In contrast with conventional meta-analysis of test accuracy studies, individual patient data (IPD) meta-analysis has the potential to establish the value of test combinations. First, in IPD meta-analysis test results can be analysed taking into account the continuous test results rather than the dichotomous classification that is generally used in reports of diagnostic and prognostic tests. The use of original continuous data instead of the dichotomized reported test results creates the possibility to detect a (gradual) relation between test result and disease and it makes it possible to estimate test accuracy at different cut-off values. Second, the additional information provided by diagnostic tests can be examined in light of the diagnostic information already known from history and clinical examination, and less expensive or less invasive tests [16,22,25-28].

Assumptions about invariance of test accuracy across a range of disease prevalences (prior probabilities) can be tested. Finally, also the association across patient-level characteristics or between patient level and study level characteristics (setting, study design) can be assessed, without the ecological fallacy problem.

To our knowledge, no IPD meta-analyses of diagnostic or prognostic research have been conducted so far. In this paper we describe the outline of a research program to systematically evaluate the potential benefits of IPD meta-analyses in the evaluation of diagnostic tests. Thereby, we selected four clinical problems from gynaecology, obstetrics and reproductive medicine that will be used as clinical cases for this methodological project:

1. Diagnosis of endometrial cancer in women with postmenopausal bleeding (PMB)

2. Prediction of preterm birth

3. Diagnosis of tubal pathology in subfertile women

4. Assessment of ovarian response in women undergoing in vitro fertilisation (IVF)

The objectives and research methods will be outlined below, and practical, methodological and clinical issues that we anticipate to encounter will be discussed.

## Objectives of the study

The major goal of this study will be the development of prediction rules and diagnostic algorithms for individual patients. We will create these rules and algorithms by performing IPD meta-analysis on the four clinical problems mentioned above. Within this major goal, we address both methodological as well as clinical objectives.

### Methodological objectives

First, we aim to contribute to the development of a framework for performing IPD meta-analyses and to provide practical and methodological recommendations on how to perform an IPD meta-analysis in diagnostic and prognostic research.

Second, we will attempt to gain a better understanding of sources of heterogeneity between studies and to explore the role of missing values in this type of meta-analysis.

Finally, we aim to compare IPD analyses with those based on aggregated data in conventional meta-analyses, to explore when the IPD approach is beneficial, and when a conventional approach suffices for reliable and unbiased estimates of diagnostic/prognostic accuracy.

### Clinical objectives

The clinical objective of the project is to create optimal diagnostic and prognostic strategies, incorporating probabilistic models for the individual patient profile and make them available to clinicians in ways that allow their practical integration with clinical practice.

With the help of IPD meta-analysis we aim to re-analyse the estimates of diagnostic or prognostic accuracy of tests in their clinical context and for different subgroups and compare them to estimates resulting from a more conventional meta-analytic approach.

Assuming that taking into account relevant patient and clinical history characteristics together with physical examination and several tests, by using probabilistic models, improves the accuracy and efficiency of the diagnostic work-up, this probabilistic approach could be used to improve clinical practice.

In addition, current guidelines for the management for each of the four clinical examples will be adjusted to reflect the results of this study and to provide support for using probabilistic models in the clinical setting.

### Clinical examples of diagnostic/prognostic problems

Prediction rules and diagnostic algorithms will be developed for each of the four clinical problems:

#### Postmenopausal bleeding

Post-menopausal bleeding (PMB) accounts for a large proportion of gynaecological consultations in both primary and secondary care [29]. In most instances, PMB results from benign causes. However, as endometrial cancer is present in 5–10% of PMB patients, further testing to exclude cancer is mandatory, but there is still controversy on the best diagnostic strategy. Currently, the first step in the diagnostic work-up of PMB is transvaginal sonography (TVS). There is debate on the value of transvaginal sonography, which could potentially be replaced by invasive investigations -hysteroscopy with or without biopsy- in some situations [30,31]. As most original studies reported the diagnostic accuracy of transvaginal sonography in a dichotomous way, they possibly have overestimated the performance of this test [23]. In addition, information gained by clinical history and physical examination (e.g. age, parity and diabetes), contains relevant diagnostic information concerning the presence or absence of endometrial carcinoma [32], which is not taken into account in the conventional meta-analysis [33]. With the individual patient data these problems can potentially be overcome [34].

#### Prediction of preterm birth

Preterm birth occurs in 7% of all deliveries – 15.000 cases per year in the Netherlands – and accounts for 70% of perinatal mortality and 40% of severe cerebral morbidity [35]. Many researchers have therefore put effort in strategies to prevent preterm birth [36]. These efforts are becoming more important, as there is now evidence that treatment with progesterone is effective in the prevention of preterm birth in high risk women. Such strategies always start with the identification of women at risk for preterm birth [37].

#### Diagnosis of tubal pathology

In the United States, about 8% of all women between 15 and 44 years are suffering from subfertility [38]. In the Netherlands, the percentage of couples suffering from subfertility is estimated to be between 12% and 17%, depending on the age of the woman [39]. With sperm defects and ovulation disorders, tubal disease ranks among the most frequent causes of subfertility. In tubal pathology, either one tube or both tubes are occluded, thus preventing the sperm to reach the oocyte. Prevalence of tubal disease has been estimated to range between 10–30%, which implicates that about 2,500 to 7,500 Dutch women are diagnosed with tubal pathology each year.

Multiple tests for the evaluation of tubal patency exist, of which the most commonly used are Chlamydia Antibody Tests (CAT), hysterosalpingography (HSG) and diagnostic laparoscopy with chromopertubation, the latter often being considered a gold standard test.

At the moment, there is no consensus on which test should be initially used in the diagnostic work-up, or on the most effective and cost-effective sequence of tests.

By using IPD meta-analysis we will integrate patient characteristics and results of diagnostic tests for individual patients with subfertility and assess various combinations and sequences of tests.

#### Assessment of ovarian response in IVF

Around 15.000 IVF/ICSI cycles are performed each year in the Netherlands. The most important single factor to determine success is maternal age. Age related decline of success is largely attributable to a progressive decrease of oocyte quality and quantity with increasing female age. Over the past two decades a number of ovarian reserve tests have been designed and evaluated for their ability to predict outcome of IVF in terms of oocyte yield and occurrence of pregnancy [40]. Many of these tests have become part of the routine diagnostic procedure in subfertile patients that will undergo assisted reproductive techniques. Based on these tests couples are counselled on their pregnancy chances prior to IVF, and individual dose adjustments are often suggested. However, assessment of mutual dependence between these tests in conventional meta-analyses is difficult and many studies report test accuracy of these continuous tests around an artificial cut-off level. Moreover, the added value of the tests to female age has hardly been addressed [41,42].

## Methods/Design

### General methods

#### Identification and selection of studies

**Figure 1:**
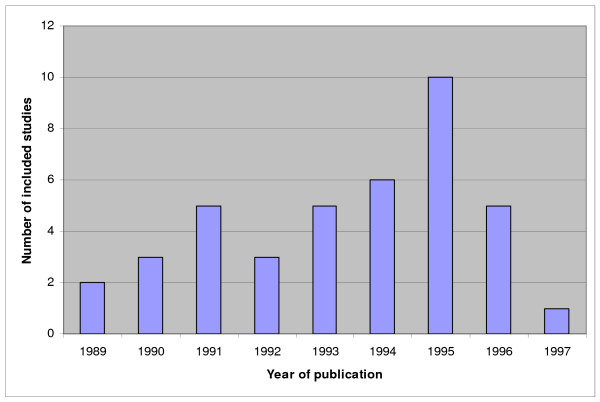
**Overview of studies included in the systematic reviews and meta-analyses on postmenopausal bleeding. Not updated**. The number of included studies is related to the year of publication.

**Figure 2:**
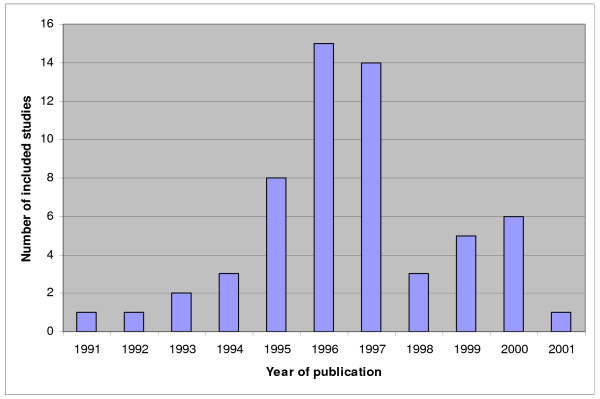
**Overview of studies included in the systematic reviews and meta-analyses on preterm birth. Not updated**. The number of included studies is related to the year of publication.

**Figure 3:**
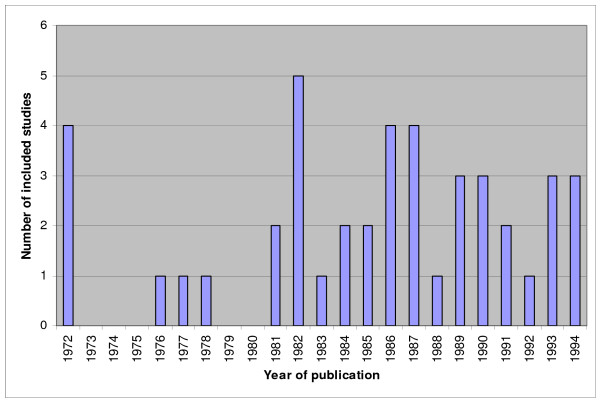
**Overview of studies included in the systematic reviews and meta-analyses on tubal pathology. Not updated**. The number of included studies is related to the year of publication.

**Figure 4:**
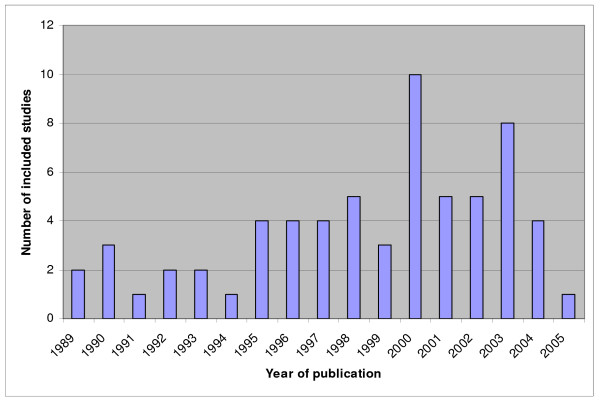
**Overview of studies included in the systematic reviews and meta-analyses on ovarian response in IVF. Not updated**. The number of included studies is related to the year of publication.

Previously, systematic reviews of studies on diagnostic and prognostic test accuracy for each of the four clinical topics were performed and by means of these reviews we identified the relevant primary research in these four areas [30,31,36,37,40,43-47]. For an overview of the amount of included studies in these meta-analyses see figures 1 to 4. We will update the performed search strategies to include studies published up to date. We will perform a computerized search, check references and asks authors of relevant studies whether they are aware of unpublished or ongoing studies. Readers of this protocol, who are familiar with studies performed on these four clinical topics that are not integrated in the previous performed meta-analyses, are also invited to approach us.

We aim to include datasets from all studies meeting the inclusion criteria of the original (updated) reviews. Studies that have met the inclusion criteria in the meta-analyses on postmenopausal bleeding were prospective studies that reported on endometrial thickness and in which the transvaginal ultrasound was performed before tissue assessment. The selection criteria for the meta-analyses on preterm birth were studies on asymptomatic or symptomatic pregnant women, cervicovaginal fetal fibronectin testing before 37 weeks' gestation, known gestation at spontaneous birth and observational cohort design. The meta-analyses on tubal pathology included studies that compared CAT or HSG to laparoscopy for tubal pathology and that described a clear distinction between tubal occlusion and peritubal adhesions. For the ovarian reserve tests meta-analyses included studies that reported on the association of follicle stimulating hormone (FSH), anti-mullerian hormone (AMH), antral follicle count (AFC), ovarian volume or clomiphene citrate challenge test (CCCT) with poor ovarian response or pregnancy after IVF. All meta-analyses only included studies with sufficient data to construct 2 × 2 tables. Exclusion criteria were a lack of binary data for constructing the 2 × 2 tables and inadequate study quality. Study quality was defined as a clear description of sampling, data collection, study design, blinding, (partial) verification and missing data. Adequate test description and description of either the population or the reference test was also included in the assessment of study quality [30,31,36,37,40,43-47].

Including all available studies in our IPD meta-analyses will maximise our ability to study the factors associated with heterogeneity in model intercepts and coefficients and diagnostic odds ratios. We will therefore also consider studies that have potentially collected relevant data, but that have been excluded in previous analyses.

#### Data acquisition

We will approach all authors of the selected original studies to inform them about this IPD meta-analysis project and invite them to share their data in this collaborative project. If they are inclined to participate, they are provided with a more detailed study proposal, and asked to send their original datasets. We ask them to send the complete database as to minimise their efforts going through their dataset to select the appropriate variables. Any data format is accepted, provided that variables and categories are adequately labelled within the dataset or with a separate data dictionary. We aim to include datasets from all studies meeting our target variables as described in table 1. Minimal requested data are (anonymous) patient identifiers, index tests and reference tests (See * in table 1). Studies in which a substantial part of these variables are missing are considered to have incomplete data. We will also ask authors to examine the provisional study list to identify any additional studies they may be aware of. In this way also data from studies that have been missed by our search criteria, or have not been published at all, will be considered for inclusion.

**Table 1:** Variables from the original studies to be included in the IPD meta-analyses.

Topics	Postmenopausal bleeding	Preterm birth	Tubal pathology	Ovarian response in IVF
Population	Postmenopausal bleeding	Asymptomatic early pregnancies	Subfertility	Indication for IVF treatment
		Threatened pre-term labour		

Patient characteristics	-Age	-Age	-Age	-Age
	-HRT use	-Obstetric history	-Fertility history	-Fertility history
	-BMI	-BMI	-PID	-BMI
	-Time since menopause	-Multiple pregnancies	-Ectopic pregnancy	-Previous ART
	-Diabetes	-Parity	-BMI	-Smoking
	-Hypertension	-Diabetes	-Pelvic surgery	
	-Use of anticoagulants			
	-Previous cancer			
	-Thyroid dysfunction			

Diagnostic tests	-TVS*	-Blood pressure	-CAT*	-FSH*
	-Hysteroscopy/curettage*	-Cervical length measurement*	-HSG*	-AFC
	-Histology of carcinoma*	-Fibronectin test*	-Laparoscopy*	-AMH

Target condition	Endometrial carcinoma*	Childs condition	Tubal pathology*	Ovarian response Pregnancy*
		Delivery prior to 32 weeks*		

Overview of variables that will be requested from the original authors.

Marked variables (*) are the minimal requested variables; studies missing a substantial part of these variables will be excluded.

Abbreviations used: HRT: Hormone Replacement Therapy, TVs: Trans Vaginal Sonography, BMI: Body Mass Index, PID: Pelvic Inflammatory Disease, CAT: Chlamydia Antibody Test, HSG: Hysterosalpingography, ART: Assisted Reproductive Therapy, FSH: Follicle Stimulating Hormone, AFC: Antral Follicle Count, AMH: Anti Mullerian Hormone.

#### Quality assessment

We will define study quality of the original studies to a large extent in the same way as it was described in the systematic reviews (see above under the heading 'data acquisition'). We will report the study quality according to the STARD statement [48]. Completeness of datasets in terms of which diagnostic indicators were assessed and to which extent data on a particular indicator are complete, together with, if possible, an assessment of how well the study execution adhered to the research protocol, will also be assessed to describe study quality. An attempt will be made to rank data sets according to their quality.

Quality of the received data will be judged by the assessment of consistency of the data and the published manuscript. We will also assess reproducibility of the reported accuracy in the manuscript using the raw data. By requesting the original research protocols we will be able to create an overview of included patients and test sequences, which might be used to explain the heterogeneity between included studies. We also will perform thorough data checks (single variables, simple tables and plots). The original investigators will be contacted to confirm missing data or to check values of doubtful validity. In addition to this, further details during discussions with primary investigators at a collaborators meeting, may shed light on specific problems encountered during study execution, and resolve differences due to the use of different definitions. Such discussions may give us more precise descriptions of the test procedures used, and the proficiency of the examiners, if the protocols were unclear on these points.

Unfortunately some data may have to be excluded from the IPD meta-analyses due to incomplete data or major inconsistencies with published results. Data are only considered to be incomplete when a substantial part of the most relevant variables was not available in the original study and the original authors are not able to provide the missing data. We emphasise that a valid diagnostic model can be derived based on fewer than all available data sets.

#### General statistical analyses

After the assessment of study and data quality the variable codes of all the acquired data will be compared between the original databases. If the variables are compatible the original data will be merged and a study identification variable will be added to reflect the stratified nature of the pooled dataset. Within this database we will create subgroups on all relevant issues concerning the clinical problems (see table 2). For all subgroups we will construct 2 × 2 tables, comparing the dichotomised test result to the final disease status. We will then calculate sensitivity and specificity, and plot the results in a ROC-space. These summary receiver operating characteristic (SROC) data and ROC curves will show the differences in the accuracy of the index tests in comparison to the best available reference tests between the different subgroups. Differences in diagnostic performance across subgroups will be accounted for using interaction effects. Furthermore, we will look at the distributions of continuous variables in both diseased and non-diseased patients in various studies. If these distributions appear to be different between studies, a correction will be applied using the multiple of the median as unit for the test result.

**Table 2:** Analyses to be performed in the IPD meta-analyses.

Topics	Postmenopausal bleeding	Preterm birth	Tubal pathology	Ovarian response in IVF
ROC analysis	-TVS*	-Cervical length*	-Age*	-Age*
			-CAT*	-FSH*
				-AFC*
				-AMH*

Univariable analyses	All relevant patient characteristics	All relevant patient characteristics	All relevant patient characteristics	All relevant patient characteristics

Multivariable model patient characteristics only	-Age	-Age	-Age	-Age
	-HRT use	-Obstetric history	-Fertility history	
	-BMI	-BMI	-PID	
	-Time since menopause	-Multiple pregnancies	-Ectopic pregnancy	
	-Diabetes	-Parity	-BMI	
	-Hypertension	-Diabetes	-Pelvic surgery	
	-Anticoagulants use	-Blood pressure		
	-Previous cancer			
	-Thyroid dysfunction			

Tests in multivariable model with tests	-TVS	-Cervical length	-CAT	-AFC
	-Hysteroscopy	-Fibronectin test	-HSG	-FSH
			-Laparoscopy	-AMH

Subgroup analysis	-BMI*		-Age*	-Age*
	-Diabetes		-PID	-Duration subfertility*
			Duration subfertility*	-Type subfertility
			-Type subfertility	-BMI*
			-BMI*	
			-CAT	

Diagnostic decision rules:				
1. Patient characteristics rule	histology if ca > 3%			
2. Selective rule	TVS if ca > 3% → > 4 mm: histology			
3. Integrated rule	TVS and histology if ca > 3%			

Decision analysis			-Patient characteristics	-Patient valuations
			-Tubal pathology	-IVF success

Combined analyses		Combination with progesterone	Combination with IVF outcome	

Overview of variables and analyses that will be used, specified for each clinical topic. Marked variables (*) will also be assessed as continuous data.

Data on continuous test results will allow us to determine different cut-off values using ROC curves and area under the curve measurements and show whether the accuracy of the test was possibly overestimated in the original studies, reporting artificial cut-off values.

After these exercises we will calculate positive and negative predicted values for the clinical problems and perform univariable analyses, using all available characteristics of clinical history, physical examination and the several diagnostic tests. The assumption of linearity between predictor and disease state will be evaluated for continuous variables using both quartile analysis and smoothed piecewise polynomials (splines) [49].

This will be followed by fitting univariable models. Subsequently, multivariable regression models will be created, both for clinical history and examination alone, as well as for various combinations and sequences of relevant patient characteristics with additional tests. This will finally lead to the development of the individual diagnostic or prognostic algorithm. We will use imputation strategies that we have applied previously for missing data at the individual level. For missing data at the study level (i.e. information not documented in a study), we will also consider imputation to allow multivariable analyses on the most complete dataset, although the added value of such major imputation efforts may be limited and will be explored in the perspective of IPD meta-analysis [50]. The multilevel approach will allow for variation in parameter estimates across studies (random effects). We will explore whether some of this study level variation can be attributed to study level characteristics, e.g. quality, design, etc. Moreover, we will assess efficiency (number of diagnostic procedures, number of subsequent procedures), and compare this to current clinical practice.

To compare the results of the included studies in the IPD meta-analysis approach we will also perform a conventional diagnostic meta-analysis for the same set of studies. As this work has in part already been performed [30,31,36,37,40,43-47] this will be a repeat of previous work, in which subtle adjustments to the methodology of previous meta-analyses will be made.

#### Model validation

To adjust for overfitting, we plan to use several internal validation techniques (bootstrap (patient level), leave one out (study-level)) [51]. We intend to internally validate the complete analytical process including the imputation of missing values and that may necessitate the writing of dedicated programs. We will also apply leave-one-out approaches, as developed in the context of the modelling of prognosis of HIV infection [52], by fitting candidate models on pooled data from all but one of the studies and testing generalisability on the omitted study. This procedure will be repeated n-1 times, rotating the left out study. We will use deviance differences to quantify the additional lack-of-fit when a model is fitted on one data set and predictions are made on another data set [53]. The deviance differences will be summed across the test studies: the best-generalizing model was that with the lowest total deviance difference. The available data-sets will also allow us to perform so called external validation. At external validation, the performance of the developed model is validated in a different data-set.

### Specific methods for clinical topics

The analyses described above will be assessed for all four clinical topics. For an outline of the individual assessments of the topics see the following part and table 2.

#### Postmenopausal bleeding

Data collected will contain patient characteristics and tests as described in table 1. Final disease status, i.e. the presence or absence of endometrial cancer, can be diagnosed with mircocurettage, curettage after dilatation and/or hysteroscopy. After univariable analysis we will build a multivariable model to predict endometrial carcinoma using the patient characteristics. Age will be defined as the age at which the first episode of postmenopausal bleeding occurred. Categorical variables with subdivisions (e.g. type and management of diabetes) will be dichotomised. We will develop two multivariable logistic regression models. The first model will be based on patient characteristics only ("patient characteristics model"). In the second model, patient characteristics will be combined with endometrial thickness as measured with transvaginal sonography ("patient characteristics and TVS model").

Since it has been reported previously that the accuracy of endometrial thickness measurement is different in obese and non-obese women and in diabetic and non-diabetic women [33], differences in diagnostic performance across subgroups will be evaluated through interaction terms.

Finally, three different diagnostic decision rules based on these two models will be explored in terms of diagnostic efficiency, and compared to current clinical practice (i.e. transvaginal ultrasound, with histological assessment in women with endometrial thickness of 5 mm or more). The three evaluated strategies will be

(1) the "patient characteristics" rule, i.e. probability estimates based on patient characteristics, and invasive diagnostics in case the probability of (pre) malignancy is over 3%. In this decision rule TVS is not performed.

(2) "selective" rule, i.e. probability estimates based on patient characteristics, TVS in case the probability for cancer exceeds 3%, and subsequent histological analyses when the endometrial thickness exceeds 4 mm.

(3) "integrated" rule, i.e. TVS in all patients, with a probability estimate based on both patient characteristics and TVS results, completed by endometrial sampling when the probability of cancer exceeds 3%.

#### Prediction of preterm birth

Data collected will contain patient characteristics and tests as described in table 1.

We will use several outcome measures, including the condition of the child. However, for the purpose of the present study, delivery prior to 32 weeks will be the primary outcome. We will look at the distribution of several characteristics, including cervical length. Subsequently, we will perform receiver-operating characteristic analysis for cervical length, as well as other continuous tests. We will build two multivariable models to predict preterm birth. The first model will be based on patient characteristics only ("patient characteristics model"). In the second model, patient characteristics will be combined with cervical length and fibronectin. We plan to combine the diagnostic data with data from the effectiveness of progesterone in the prevention of preterm birth, as the latter agent has found to be effective in the prevention of preterm birth in women with a previous preterm delivery [54]. By doing so, we can assess the efficiency of several strategies to prevent a preterm birth.

#### Diagnosis of tubal pathology

Data collected will contain patient characteristics and tests as described in table 1.

Presence of tubal pathology will be the primary outcome measure. We will perform all analysis twice. In the first analysis, tubal pathology will be defined as two-sided tubal occlusion. In the second analysis, tubal pathology will be defined as any form of tubal occlusion, be it one-sided or two-sided. We will perform ROC-analyses for continuous variables, such as age and CAT. Subsequently, univariable logistic regression analysis will be performed. This analysis will continue on an analysis that we have performed previously [55]. Again, we will develop several multivariable models. The first model will be based on patient characteristics only. In a second model, these patient characteristics will be combined with the Chlamydia Antibody Test measurements. We can also use various combinations and sequences of patient characteristics and additional tests. These models will lead to the development of the diagnostic algorithm for the individual patient suffering from tubal pathology. Finally, the data of the constructed algorithms for tubal pathology will be combined with data on the prediction of successful IVF-outcome.

#### Assessment of ovarian response in IVF

Data collected will contain patient characteristics and tests as described in table 1.

For the analyses on the ovarian reserve tests we will use two outcome measures; ovarian response and pregnancy. The exact definition of these two outcome measures will depend on the available data and on the outcome of the discussion at the initiating collaborative work-shop. Variables considered are shown in table 1. As for the other clinical examples, ROC-analysis will be performed. We will develop models for female age alone and for female age plus AFC. As AFC is at present found to be the best predictor for IVF outcome, we plan to compare models with the other tests to a model based on female age plus AFC.

We have previously published a decision analysis in which we integrated patient valuations of subfertile couples (incorrect withholding of IVF versus undergoing IVF without success) and predicted probabilities of IVF-success. This analysis revealed a so-called threshold ROC-curve, which showed the minimal accuracy that an ovarian test (or combination of tests) should have to be of clinical value [50]. We will repeat the analysis using the data obtained from the original studies.

### Implementation of probabilistic approach in clinical practice

We have developed a website with information on the progress of the project. See http://www.ipd-meta-analysis.com/ipd. The website will contain protocols, including the description of the objectives of each project and proposals for the statistical analyses. Moreover, the diagnostic algorithms that will be the result of the project will be available from the website after the studies have been completed.

The clinical "end products" of these IPD meta-analyses will be prediction rules for each of the four clinical problems: women with PMB, women at risk for preterm birth, women suspected of having tubal pathology, and women starting with IVF. The results will be made available through simple scoring chards as well as logistic regression models. The latter will become accessible through web applications at which doctors can enter relevant data of the patient. Furthermore, such prediction rules will be made available for patients, as we did previously with prediction rules developed for spontaneous pregnancy in subfertile couples [56]. We will do this with score forms on paper, website applications and software available through personal digital assistants.

### Collaborative work-shop and definitions

Workshops will be organised with all investigators of the included studies. In addition to discussing the IPD-meta-analysis project in general, as well as the practical, methodological and data-related aspects of each original study, these meetings are also important to build trust. During these workshops, we will discuss and refine the study protocol, examine patient characteristics and information from diagnostic tests that are to be analysed, the data checking procedures and the main analyses to be performed. Criteria for classifying test results, including results of reference tests, as positive or negative will also be discussed, taking into account that the exact nature of tests and procedures will differ between studies and centres. We will also propose a timetable and a publication policy, including a list of anticipated publications, with a collaborative group authorship for these publications, to be discussed and agreed upon by all collaborating authors.

### Publication policy

The results from the IPD meta-analysis will be presented at a collaborators meeting. Any subsequent articles on the results of the meta-analysis will be published under the name of the collaborative group. It will also be circulated to the collaborators for comments, amendments and approval before finally being submitted. In the case of any disagreement, the following fundamental principle will be applied; the report should provide the meta-analysis results, presenting all of the available evidence, but will not include any interpretations of the data, except those that are unanimously decided upon by all collaborators. Any collaborating group is free to withdraw its data at any stage.

## Discussion

Although it is at present stage not possible to exactly anticipate on the clinical and methodological results from the planned steps in each of the four clinical topics, we expect to have the following knowledge available at the end of the project:

Methodological knowledge:

• Differences between conventional meta-analyses with summary estimates of sensitivities, specificities and ROC-curves, and IPD meta-analyses.

• Knowledge of quality of reporting on individual studies

• Knowledge of completeness of data and ways to deal with missing values

• Knowledge of differences and similarities in distributions of parameters between studies

Clinical knowledge:

• Prediction models and diagnostic models obtained with IPD meta-analyses and the relative performance in comparison to aggregate meta-analyses

• Estimates of accuracy and calibration of the prediction models and diagnostic models

• Integration of the diagnostic and prognostic knowledge with knowledge of therapeutic effectiveness

Increased efficiency of the diagnostic work-up by making optimal use of the patient characteristics combined with the results of the diagnostic tests, will probably decline the need of invasive tests and contributes to improved patient care. With help of the results of the four clinical problems, we can then assess the potential value of IPD meta-analysis in diagnostic and prognostic models, compared to conventional diagnostic meta-analysis.

From the experiences in the present proposal, we will provide recommendations on how to perform IPD meta-analysis in prognostic and diagnostic research.

A final step in the work-plan is to provide these data through the internet. The progress of the project can be followed on http://www.ipd-meta-analysis.com/ipd.

## Competing interests

The authors declare that they have no competing interests.

## Authors' contributions

BM is the principal investigator of the study described in this article. BM, LB, KK and GR developed the initial study protocol. KB and BO participated in the study design and coordination. KB wrote the first draft of the manuscript. All other authors commented on this draft and contributed to the final manuscript.

## Pre-publication history

The pre-publication history for this paper can be accessed here:

http://www.biomedcentral.com/1471-2288/9/22/prepub
